# Increased Oxidative Stress as a Selective Anticancer Therapy

**DOI:** 10.1155/2015/294303

**Published:** 2015-07-26

**Authors:** Jiahui Liu, Zhichong Wang

**Affiliations:** State Key Laboratory of Ophthalmology, Zhongshan Ophthalmic Center, Sun Yat-sen University, 54 Xian Lie Nan Road, Guangzhou 510060, China

## Abstract

Reactive oxygen species (ROS) are closely related to tumorgenesis. Under hypoxic environment, increased levels of ROS induce the expression of hypoxia inducible factors (HIFs) in cancer stem cells (CSCs), resulting in the promotion of the upregulation of CSC markers, and the reduction of intracellular ROS level, thus facilitating CSCs survival and proliferation. Although the ROS level is regulated by powerful antioxidant defense mechanisms in cancer cells, it is observed to remain higher than that in normal cells. Cancer cells may be more sensitive than normal cells to the accumulation of ROS; consequently, it is supposed that increased oxidative stress by exogenous ROS generation therapy has an effect on selectively killing cancer cells without affecting normal cells. This paper reviews the mechanisms of redox regulation in CSCs and the pivotal role of ROS in anticancer treatment.

## 1. Introduction

Reactive oxygen species (ROS) is a collective term for oxygen-containing chemical species that are converted directly or indirectly from free oxygen but are more chemically reactive [1]. Low to moderate levels of ROS are indispensable to normal cellular proliferation, differentiation, and survival [2]. Some reports have shown that the addition of low concentrations of superoxide or hydrogen peroxide (10 Nm–1 *μ*M) to the culture medium is effective for stimulating the growth of hamster and rat fibroblasts* in vitro* [3, 4]. In general, systems in aerobic organisms are developed to modulate the content of ROS by balancing the generation and scavenging of ROS within a nontoxic range. But once the balance is broken, cells suffer from oxidative stress. Rapid increases in intracellular ROS may lead to cellular transformation and tumorigenesis. For example, researchers have found in BHK-21 cells that deaths of apoptotic cells become obvious after the exposure to 10–100 *μ*M hydrogen peroxide [5]. Substantial growth of ROS brings cells irreversible damage and finally kills them. However, it is just these biological features of ROS that make it possible to be used to kill tumor cells [2].

Since Lapidot discovered leukemia stem cells in 1994, researchers have shed light on the study of cancer stem cells (CSCs) [6]. CSCs are defined by their capacity to self-renew and differentiate into heterogeneous nontumorigenic cancer cell types in accordance with their microenvironment and the status of the whole body [7, 8]. CSCs, which only account for about 0.05%–1% of the whole tumor cell population [9–11], play an important role to tumor formation and development. They are believed to relate closely to chemo- and radioresistance and disease recurrence [12–16]. Therefore, CSCs are considered as good targets for cancer therapy [17]. The study of intracellular ROS in CSCs remains an attractive field for research. Little is known about the biological effects and regulatory mechanism of ROS in the CSC subpopulation. This review focuses on ROS's regulation effect on CSCs and the therapeutic effect on cancer eradication.

## 2. Lower ROS Production in CSCs

ROS are mainly composed of free radicals such as superoxide (O_2_^−^), hydrogen peroxide (H_2_O_2_), and hydroxyl radical (HO^∙^), which contain oxygen and peroxides tending to form radicals [1]. Under physiological conditions, O_2_^−^ is principally generated as a consequence of incidental electron leakage from the mitochondrial electron transport chain (ETC) [18] and is usually immediately converted into H_2_O_2_ by mitochondrial Mn-containing superoxide dismutase (MnSOD, SOD2), cytosolic Cu/Zn-containing SOD1, or extracellular SOD3 [19, 20]. H_2_O_2_ can be catalyzed to release highly toxic HO^∙^ by gaining an extra electron or be scavenged by the reaction of glutathione peroxiredoxin (Prx), peroxidase (Gpx), or catalase [21–23]. Besides the mitochondrial mechanism, ROS can also be generated by the NADPH oxidase complex (NOX), cyclooxygenase (COX), cytochrome c oxidase, and xanthine oxidase (XO) [24].

The production of cellular ROS must go with scavenging of ROS. The currently available body of evidence shows that powerful scavenger systems, which are mainly divided into two classe: antioxidant enzyme system and sulfur reduction buffer system, can maintain the intracellular ROS at low levels. The former class contains the superoxide dismutase family (SOD1, SOD2, and SOD3), catalase (CAT), and peroxidase. In addition, ascorbic acid and vitamin E are also involved and play vital roles in antioxidant enzyme system [25]. The latter class contains reduced glutathione (GSH), thioredoxin (TRX), and thioredoxin reductase (TRXR). Glutathione peroxidase converts H_2_O_2_ to H_2_O and O_2_ through coupling with the transformation of GSH to oxidized glutathione [2]. In general, GSH and GSSG maintain homeostasis within cells, and their contents have become important indicators of the antioxidant capacity of cells [26].

Cells maintain redox homeostasis, which is favorable for organisms, through a balance of generation and elimination of ROS. Aerobic organisms actively use ROS in signal transduction pathways to regulate cell proliferation, differentiation, and survival, in a way of interacting with macromolecules by reversible oxidative modifications [27–30], while excessive amounts of ROS, regardless of their source, cause irreversible peroxidation of nucleic acids, lipids, amino acids, and carbohydrates, resulting in cellular senescence, apoptosis, or transformation and triggering a series of pathological processes, such as cardiovascular diseases, neurodegenerative diseases, aging, and cancer [31–36]. For example, ROS induce nuclear DNA mutations by activating oncogene or inactivating tumor suppressor genes and damage nuclear DNA repair mechanisms, resulting in the generation of tumor-initiating cells. Persistent ROS-induced oxidative stress expands the clonal selection of these cells, gradually making them form subsets with new features. Tumor then occurs as a consequence of reduced apoptosis and increased genomic instability and heteromorphism [37, 38].

Compared to differentiated cells, normal stem cells are more glycolytic to reduce more oxidative damage due to ROS [39–41]. Similarly, CSCs present lower energy metabolism rate and produce less ROS compared with non-CSCs [17, 42]. This can be achieved by a combination of mechanisms that is unique to a given tumor, such as (a) upregulation of ROS scavengers, (b) downregulation of ROS-producing enzymes, (c) promotion of glycolysis, (d) reduced mitochondrial mass, and (e) low oxygen consumption [43–46]. As evidenced by Ishimoto et al.'s study, the CSCs of human gastrointestinal tract improve abilities of GSH synthesis with a cystine-glutamate exchange transporter in order to enhance its defensive performance against ROS [47].

## 3. ROS Activates HIFs in CSCs

The morphology, metabolism, and proliferation of CSCs are critically dependent on their microenvironment. CSCs will not only strive for adapting to their microenvironment but also actively create their preferable niche. Hypoxia is an essential feature of the tumor microenvironment because of the chaotic vasculature and poor oxygen diffusion in solid cancers. The normal oxygen tension in healthy tissue is approximately 7% (53 mmHg), but this tension can show differences in tumors depending on the level of hypoxia, that is, from physiological (~7%) to severe (<1%) hypoxia [48]. It has become increasingly clear that a hypoxic microenvironment is beneficial for the maintenance of CSCs in virtually all tissues of the body [49–51]. For example, in glioblastomas, hypoxia sustains the undifferentiated state of CSCs by elevating some important stem cell markers, such as Oct4 and Sox2, slows down their growth to the quiescent stage, and increases their colony-forming efficiency and migration [52]. Besides, since hypoxia potentiates the CSC-mediated inhibition of T cell proliferation and activation in glioma and further inhibits macrophage phagocytosis compared with normoxia conditions, immunosuppression can be reinforced [53]. Hypoxia also improves CSCs' abilities of invasion and resistance against therapy, which poses a challenge to anticancer therapeutics [54].

In hypoxic environment, elevated ROS can activate hypoxia inducible factors (HIFs) [48]. HIFs are a type of heterodimers made up from HIF-1*α*, HIF-2*α*, or HIF-3*α* bound to HIF-*β*/ARNT (aryl hydrocarbon receptor nuclear translocator). While HIF-*β* is constitutively and ubiquitously expressed among many cell types, all HIF-*α* subunits are regulated by intracellular oxygen sensors, which are referred to as prolyl hydroxylate enzymes and asparaginyl hydroxylase [55]. Under normoxic conditions, the von Hippel-Lindau (VHL) E3 ligase complex targets HIF-*α* subunits for proteasomal degradation [56]. Under decreased oxygen level, HIF-*α* subunits can be stabilized through the activation of intracellular signaling pathways by ROS. Take two simple examples. Activation of the PI3K-AKT-mTOR pathway can promote the synthesis of HIF-*α*, while inhibition of hydroxylase activity can prevent HIF-*α* degradation [55, 57, 58]. A wealth of evidence illustrate that HIFs induce metabolic reprogramming from oxidative phosphorylation to anaerobic glycolysis as well as lactic acid fermentation, by activating lactate dehydrogenase A and phosphorylating the E1*α* subunit of pyruvate dehydrogenase. This metabolic reprogramming is widely accepted as a hallmark of cancer because it can not only earn more ATP for cancer cells but also reduce the cytotoxic ROS levels in order to resolve the energy crisis within vigorous tumors and to help cancer cells survive the state of hypoxia. Additionally, it also enhances resistance to chemotherapy and radiotherapy treatments [59, 60]. HIF-2*α* promotes the expression of multiple antioxidant enzymes and DNA damage repair enzymes, thereby reducing the intracellular ROS levels and limiting the accumulation of DNA damage [61–63]. Only under hypoxia (1%) can HIF-1*α* be stabilized, and a low activity is observed at 5% O_2_ (resembling the end-capillary oxygen conditions) [64]. However, HIF-2*α* is stabilized at a wider range of oxygen tensions, ranging from severe hypoxia (<1% oxygen) to more physiologically relevant tension in tumors (2–5% oxygen) [64, 65]. HIF-1*α* and HIF-2*α* are highly homologous and bind to similar hypoxic response elements [56]. However, due to their unique target genes and expression patterns, they have their individual biological roles. Li et al. found that, under severe hypoxic conditions, HIF-2*α* is markedly expressed only in glioma stem cells but not in nonstem cells, whereas HIF-1*α* exists in both tumor subpopulations. It has also been suggested that the HIF-2*α*-mediated upregulation of Oct4, Glut1, SerpinB9, and VEGF may facilitate CSCs in metabolism, proliferation, survival, and escape from immune surveillance [65]. As evidenced in a number of various tumors in recent studies, hypoxia initially induces a transient activation of HIF-1*α* followed by persistent HIF-2*α* expression occurring after more prolonged periods of hypoxia and this hypoxic switch to HIF-2*α* can enhance the CSC population [66–68]. The expression of HIF is associated with the poor survival of patients with cancer [69].

Early CSC model proposed that CSCs are the driving force of tumorigenesis due to their abilities of self-renewal and irreversible multilineage differentiation through either asymmetric or symmetric cell division. In comparison, their offspring, namely, progenitor cells and differentiated cancer cells, no longer possess tumorigenic potential [70]. Therefore, anticancer treatment targeting CSCs holds great promise [24]. However, recently emerging lines of evidence have revised the earlier model to a dynamic model. It is now suggested that CSCs and non-CSCs can be bidirectional converged, an effect that is governed by their microenvironment; that is, progenitor cells and differentiated cells can reacquire their self-renewal capacity through reprogramming into CSCs [12, 71–75]. HIFs are considered as crucial regulators of the stem cell phenotype through Notch signaling pathways and induce the expression of stem cell markers, such as Oct4 [65], and induce pluripotent stem cell (iPSC) factors, Oct4, Nanog, Sox2, Klf4, and c-Myc, in many cancer cell types, including prostate, brain, kidney, cervix, lung, colon, liver, and breast tumors [76]. Similarly, multiple types of CSC-specific cell-surface markers, including CD133, CD44, and VEGF-A, can be markedly upregulated by HIFs [77–79], modulating the self-renew potential of CSCs. Studies by Heddleston et al. have elucidated that HIF-2*α* can reprogram the nonstem population of gliomas into CSCs through the upregulation of important stem cell factors, such as Oct4, Nanog, and c-Myc [80]. Consequently, it is necessary to evolve current cancer treatments to target both bulk differentiated cells and CSCs in tumor.

## 4. Regulation of ROS through CSC Markers in CSCs

CSCs can be identified and isolated primarily by CSC-specific cell-surface marker expression (Table 1). It has been demonstrated that CD34, CD133, CD44, and ALDH1 mark CSCs in leukemia and some solid tumors [81–86]. Nonetheless, there is a lack of a universal expression of surface markers to identify CSCs in all types of tumors [17]. As mentioned above, ROS-induced HIFs can enhance the expression of a variety of CSC biomarkers. For instance, insulin-like growth factor binding protein 3 (IGBP3) can suppress ROS-mediated cytotoxicity by novel insulin-like growth factor (IGF), independent antioxidant activity, thereby increasing CD44H cells in esophageal squamous cell carcinoma (ESCC) and facilitating ESCC cell adaptation and survival under hypoxia [87]. Interestingly, the biomarkers can modulate the level of ROS via different mechanisms.

It is well known that CD13, which is a marker of liver CSCs, is associated with tumor invasion, angiogenesis, and antiapoptosis [88, 89]. CD13 is a negative ROS regulator that results in the inhibition of apoptosis and the enhancement of the stemness of CSCs [24]. As a consequence, the survival of liver CD13^+^ CSCs in hypoxic lesions after chemotherapy contributes to the high expression of aminopeptidase N, a ROS scavenger enzyme. In addition, immunohistochemical analyses have indicated that CD13 coexists with N-cadherin in surviving cancer cells [89]. Gclm, which encodes a glutamate-cysteine ligase that catalyzes the rate-limiting step in the synthesis of GSH, is overexpressed in the CD13^+^CD90^−^ fraction in PLC/PRF/5 cells, the ROS level of which is lower than that of the CD13^−^ population. Both the CD13-neutralizing antibody and CD13 inhibitor ubenimex can stimulate ROS production, increasing the ROS concentration to that found in CD13^−^ cells. In mouse xenograft models, the administration of ubenimex attenuates the self-renewal and tumor-initiation potentials of CD13^+^ cells [90]. Therefore, CD13-neutralizing antibody or other inhibitors can kill CSCs effectively.

As a cellular adhesion molecule for hyaluronic acid, CD44 is the most prevalent CSC molecular marker [91–93] and is widely expressed in multiple tumors, including breast cancer [94], head and neck squamous cell carcinoma [95], pancreatic cancer [96], colorectal cancer [97], and prostate cancer [83]. Its variant isoform, CD44v, can interact with and stabilizes xCT, a subunit of a glutamate-cystine exchange transporter located at the plasma membrane, thereby promoting cystine uptake for GSH synthesis and contributing to ROS defense [83, 97], and this finding was validated by the study conducted by Ishimoto et al. on gastrointestinal CSCs [47]. These researchers recently forced the expression of miRNA-328 in gastrointestinal cancer cell lines and found that CD44 expression was reduced, resulting in the repression of cancer cell growth* in vitro* and* in vivo*, and impaired resistance to ROS [98]. CD44 thus maintains a key role in the GSH-dependent antioxidant system in cancer cells [99].

CD138, which is also called syndecan-1, belongs to the mammalian syndecan family of heparin sulfate proteoglycans. Shimada et al. obtained holoclones harboring the biological properties of stemness from single-cell cultures of the PC3 human prostate cancer cell lines. Syndecan-1 is overexpressed in these holoclones and downregulates the expression of NADPH oxidase, thereby decreasing ROS generation. The* in vitro* silencing of syndecan-1 strongly destabilizes the holoclones by increasing ROS production, whereas* in vivo* syndecan-1 deficiency lowers the frequency of primitive cells expressing stem cell markers and markedly represses cancer propagation. It has also been discovered that a high level of Bcl-2 in holoclones is tightly linked to syndecan-1 [167]. This finding is consistent with Lagadinou et al.'s discovery that the overexpression of Bcl-2 in acute myelogenous leukemia CSCs maintains the ROS synthesis rates at low levels and stabilizes the primitive cells in a quiescent state [168]. However, in Waldenstrom's macroglobulinemia, a lower ROS level is detected in CD20^−^CD138^−^ cells compared with CD20^+^CD138^−^ cells and CD20^+^CD138^+^ cells [169].

## 5. ROS Plays a Key Role in Anticancer Therapy

The Warburg effect refers to the phenomenon that cancer cells gain energy primarily from glycolysis even under aerobic conditions, leading to increased ROS production [170]. Although the ROS levels, which are counteracted by elevated antioxidant defense mechanisms in cancer cells, are compatible with cellular biological functions, they are still higher than those observed in normal cells. Cancer cells may be more sensitive than normal cells to the accumulation of ROS, which offers an interesting therapeutic window [43]. Hence, directly increasing ROS to reach a threshold that is incompatible with cell viability and targeting the enhanced antioxidant mechanisms can selectively kill cancer cells, without affecting normal cells [42, 171]. Despite the low level of ROS in CSCs and the active ROS detoxifying systems, elevating the concentration of ROS still has the ability to eliminate CSCs. The anticancer therapies by regulating ROS levels are shown in Table 2.

Many antineoplastic chemotherapeutic agents, including taxanes, vinca alkaloids, and platinum coordination complexes, are currently used to induce high levels of ROS, resulting in cell death [189–191]. Paclitaxel, a mitotic inhibitory drug, can stabilize microtubules and therefore interfere with the normal breakdown of microtubules during cell division. In breast cancer cells, paclitaxel causes the translocation of Rac1, which positively regulates the activity of NOX, thereby promoting ROS generation. Paclitaxel-induced ROS is accumulated mainly outside the cell, provoking lethal damage to bystander cancer cells not exposed to paclitaxel, whereas the intracellular ROS levels remained unchanged [174]. The tumor suppressor promyelocytic leukemia protein (PML) has been demonstrated to play a critical role in the maintenance of quiescent chronic myeloid leukemia (CML) stem cells. Arsenic trioxide, which can induce ROS production and PML degradation, is introduced to eradicate CML stem cells [172]. Niclosamide, a potent antineoplastic drug that inactivates the NF-*κ*B pathway and increases the ROS level, can preferentially kill progenitor/stem cells from acute myelogenous leukemia (AML) patients but spare those from normal bone marrow [175]. Consistent with this finding, parthenolide induces the apoptosis of CSCs in AML and blast crisis CML through mechanisms involving the inhibition of NF-*κ*B and the proapoptotic activation of p53 and elevated ROS levels, which are likely obtained by a high level of myeloperoxidase [188].

At present, radiotherapy is widely used in various types of cancer treatments, mainly depending on ROS. Water radiolysis occurs in an extremely short period of time (~10^−8^ s) after ionizing radiation [192]. Several hours after exposure, intracellular ROS from biological sources are greatly enhanced [193]. Increased ROS generation in human lung carcinoma A549 cells exposed to X-irradiation is accompanied by an enhancement of the mitochondrial membrane potential, a promotion of mitochondrial respiration, and the maintenance of the ETC enzyme activities [194]. Furthermore, mitochondrial dysfunction and upregulation of NOX resulting from ionizing radiation contribute to persistent oxidative stress [176, 195]. Tumor recurrence after radiation is attributed to preferential activation of the DNA damage checkpoint response and increases the DNA repair capacity and antioxidant defense [196, 197]. However, discrepant observations are provided by the recent finding that CSCs are more radiosensitive than non-CSCs [198, 199]. Such contradictory results may be due to the limited experimental techniques or the dynamic characteristics of CSCs, which require further clarification.

An enhanced GSH concentration within cancer cells appears to be actively involved in mechanisms of chemoradioresistance [200]. Due to the high content of GSH, tumor cells are more sensitive to drugs affecting GSH metabolism than normal cells [171]. Glutamate-cysteine ligase (GCL), as the rate-limiting enzyme in GSH synthesis, has been targeted in anticancer therapy. Buthionine sulfoximine (BSO), which can inhibit GCL activity, is the only clinically used drug to suppress* de novo* GSH synthesis [180]. In addition, sulfasalazine, an anti-inflammatory drug with specific xCT inhibitory activity, markedly reduces the cystine uptake, GSH level, and growth and viability of human pancreatic cancer cells and chronic lymphocytic leukemia cells both* in vitro* and* in vivo* [181, 182].

With the exception of GSH, thioredoxin is also an important component of intracellular redox systems. Auranofin is a gold-containing compound that functions as an antirheumatic drug and a thioredoxin inhibitor. Apoptosis is accompanied by an increased generation of H_2_O_2_ in ovarian cancer cells, which reflects the importance of the thioredoxin metabolism in tumor cell survival [187]. The simultaneous administration of auranofin and BSO has been validated to induce oxidative stress and clonogenic killing in human head and neck squamous carcinoma cells and to increase their sensitivity to epidermal growth factor receptor inhibitors [201].

In most cases, combined therapy lends credence to the success of cancer treatment. To cite some effective examples, ionizing radiation combined with arsenic trioxide improves the therapeutic efficacy in human prostate cancer cells because the combined treatment results in enhanced ROS production compared with each individual treatment, thereby provoking autophagy and apoptosis by inhibition of the Akt/mTOR signaling pathways [202]. Mechanistically, the sulforaphane and imatinib combined treatment can kill CD34^+^CD38^−^ leukemia stem cells by inducing ROS production and decreasing the GSH level [203]. The combination of a CSC marker inhibitor with ROS-inducing therapy, such as chemo- and radiotherapy, can eradicate CSCs and thereby annihilate the whole tumor. In mouse xenograft models, the combination of a CD13 inhibitor with 5-fluorouracil therapy improves the treatment of liver cancer [90].

Because ROS are a central contributor in tumor occurrence and progression, antioxidants should prevent tumorigenesis through the elimination of excessive ROS and restoration of the redox balance [204]. In addition to their protective role as preventive reagents against cancer, there is evidence that antioxidant supplementation during chemotherapy presents promising potential to reduce dose-limiting toxicities [205]. A large epidemiologic study (including 132,837 women and men) conducted in China demonstrated that vitamin E intake, either from diet or supplements, may reduce the risk of liver cancer [206]. The opposite result was found in another large study conducted in England, which revealed that the combination of *β*-carotene and vitamin A had no benefit and may have an adverse effect on the incidence and mortality of lung cancer [207]. In a two-stage mouse skin carcinogenesis model, a diet consisting of Nrf2 activators against oxidative stress significantly decreased the incidence of skin tumors [208].* In vitro* experiments have proven that some nutrients with antioxidant characteristics, that is, vitamins A and D, genistein, (-)-epigallocatechin-3-gallate, sulforaphane, curcumin, piperine, theanine, and choline, can modify the self-renewal capacity of CSCs [209]. That being said, further research is needed to confirm the utility of antioxidants in cancer prevention. The titration of the amount of antioxidant should be strictly controlled because too-high or too-low levels may promote tumor survival and development.

## 6. Conclusion

CSCs have evolved to maintain a low level of intracellular ROS as a consequence of the modulation of redox systems, metabolic reprogramming, and reduced mitochondrial DNA levels. Increasing evidence proposes that a low concentration of ROS can maintain the stemness of CSCs and contribute to tumorigenesis and development. Therefore, HIF stabilization induced by ROS in CSCs plays a critical role. However, the underlying mechanisms in these processes remain to be elucidated in depth. There exist various systems for the regulation of ROS in CSCs, such as through CSC surface markers, and these help maintain the ROS at a favorable level. Consequently, the induction of oxidative stress appears to be a promising approach for the preferential killing of cancer cells, including CSCs. In addition, a more detailed investigation of therapies with direct or indirect effects on ROS will help define a made-to-order therapeutic schedule with a lower tendency toward promoting the development of resistance to treatment.

## Figures and Tables

**Table 1:** Surface markers of cancer stem cells in different types of tumors.

Tumor type	CSC markers	References
AML	CD34^+^/CD38^−^, CD44^+^, CD34^+^/CD123^+^, CD47^+^	[6, 100–104]
ALL	CD34^+^/CD10^−^, CD34^+^/CD19^−^	[105]
Bladder cancer	EMA^−^/CD44v6^+^, 67LR^+^/CD66c^−^, CD44^+^/CK^5+^/CK20^−^	[106–108]
Breast cancer	Lin^−^/CD44^+^/CD24^−/low^/ESA^+^, ALDH1^high^, CD55^high^, CD44^+^/CD49f^high^/CD133/2^high^, CD176^+^	[94, 109–112]
Cervical cancer	CD44^high^/CD24^low^, CD49f^+^/CD133^low^	[113, 114]
Colorectal cancer	CD133^+^, CD44^+^/EpCAM^+^, CD44^+^/CD166^+^, CD24^+^/CD29^+^, ALDH1^high^, Lgr-5^+^	[97, 115–119]
Esophageal cancer	CD271^+^, CD44^+^/CD24^−^, CD90^+^	[120–122]
Gallbladder carcinoma and cholangiocarcinoma	CD44^+^/CD133^+^, CD24^+^/CD44^+^/EpCAM^high^	[123, 124]
Gastric cancer	CD44^+^/CD54^+^, CD90^+^	[125, 126]
Glioma	Podoplanin^+^, CD15^+^, A2B5^+^, CD44^+^, CD133^+^	[127–131]
Head and neck squamous cell carcinomas	CD44^+^, ALDH^high^	[95, 132]
Liver cancer	CD90^+^/CD44^+^, CD133^+^/CD44^+^, EpCAM^+^, CD176^+^, CD13^+^,	[90, 112, 133–136]
Lung cancer	CD133^+^, CD44^+^, CD176^+^, CD56^+^, CD90^+^, CD166^+^	[112, 137–140]
Melanoma	ABCB5^+^, CD271^+^, JARID1B^+^, CD133^+^, CD20^+^	[141–145]
Nasopharyngeal cancer	CD44^+^, ALDH1^+^, CD133^+^	[146–148]
Oral squamous carcinoma	CD133^+^, CD44^+^/SSEA-4^+^	[149, 150]
Osteosarcomas	CD44^+^/CD105^+^/Stro1^+^, CD117^+^/Stro-1^+^, Nes^+^/CD133^+^, ABCA5^+^	[151–154]
Ovarian cancer	CD133^+^, CD44^+^, CD117^+^, CD44^+^/CD24^+^, CD105^+^	[155–159]
Pancreatic cancers	CD44^+^/CD24^+^/ESA^+^, CD133^+^, CXCR4^+^	[96, 160]
Prostatic cancer	CD44^+^/Integrin*α*2*β*1^high^/CD133^+^, Sca-1^+^, PSCA^+^, CD10^+^, CD164^+^	[83, 161–164]
Skin squamous cell carcinoma	CD34^+^, Integrin*α*5*β*1^high^	[165, 166]

EMA: epithelial membrane antigen; 67LR: 67 kDa laminin receptor; ESA: epithelial special antigen; ALDH1: aldehyde dehydrogenase 1; EpCAM: epithelial cell adhesion molecule; Lgr-5: leucine-rich-repeat containing G-protein-coupled receptor 5; JARID1B: the H3K4 demethylase; Sca-1: stem cell antigen-1; PSCA: prostate stem cell antigen.

**Table 2:** Anticancer therapies according to their different mechanisms in regulating ROS levels.

Mechanism	Therapy	Reference
Generation of ROS		
Mitochondrial respiratory chain	Arsenic trioxide, anthracyclines (doxorubicin, daunorubicin, or epirubicin)	[172, 173]
NOX	Paclitaxel, ionizing radiation, niclosamide, AGX-891, AG-221	[174–178]
COX	Celecoxib	[179]
Elimination of ROS		
GSH	Buthionine sulfoximine, sulfasalazine, NOV-002, 6-anicotinamide, l-asparaginase, small molecule 968	[180–186]
GSSH	Auranofin	[187]
Myeloperoxidase	Parthenolide	[188]
